# Laparoscopic management of a primary gallbladder hydatid cyst with daughter cysts in the common bile duct: Case report

**DOI:** 10.1016/j.amsu.2022.104165

**Published:** 2022-07-12

**Authors:** Ranim Kazzaz, Dana Nashed, Ghaith Izz Aldeen Sattout, Nourelhuda Issa, Ahmad Aldakhil, Ousama Bitar, Aghyad Kudra Danial

**Affiliations:** a Faculty of Medicine, University of Aleppo, Syria; b Ousama Bitar, General Surgeon, Private Aleppo Hospital, Syria; c Aghyad Kudra Danial, General Surgeon, Department of Surgery, Faculty of Medicine, University of Aleppo, Syria

**Keywords:** Gallbladder, Primary hydatid cyst, Laparoscopic management, Common bile duct, Daughter cyst

## Abstract

**Introduction and importance:**

Primary Gallbladder hydatid cysts are a very rare phenomenon caused by Echinococcus granulosus. Hydatid cysts usually present as hepatic or pulmonary lesions, but in our case, it presented in the gallbladder with three symptomatic daughter cysts in the CBD. Echinococcus caused by E.granulosus Is the most common parasite causing the disease, accounting for 95% of the cases.

**Case presentation:**

75 - year - old male presented with colic pain, jaundice, itching, vomiting, nausea, insomnia, Positive murphy's sign, and elevated AST and ALT. Total bilirubin was also elevated with no hepatomegaly or splenomegaly.

**Clinical discussion:**

This disease is endemic in Mediterranean countries due to high contact with the host of the parasite the lumen or on the external surface of the gallbladder. Which can come from the portal system or the spreading of brood capsules through the biliary tract.

**Conclusion:**

The method of investigation used was USG, CT, ERCP which are affordable and accessible in low-income countries including Syria. we would like to highlight this rare presentation and the possibility of using laparoscopic surgery.

## Highlights

• Primary hydatid cyst is usually found in the liver, lungs and brain in our case it presented in the gallbladder and common bile duct which is very rare.
• We can encounter primary hydatid cyst in unexpected organs especially in countries with high prevalence.
• Jaundice in our case was the main presenting symptom.
• The role of laparoscopic surgery in treatment because of its less invasive nature and the low spillage possibility. This is the second case treated via laparoscopic surgery.

## 1 Introduction

*Hydatidosis*, a disease caused the larval stage of Echinococcus granulosus, a ∼5–10 cm adult tapeworm, has two forms (cystic echinococcosis, alveolar echinococcosis) [1].

Human cystic echinococcosis caused by E-granulosus is the most common accounting for more than 95% of the estimated 2–3 million annual worldwide cases [2].

The ingestion of the eggs, excreted in the feces of infected animals, causes the infection [3].

It typically results in the formation of a hydatid cyst, frequently in the liver or the lungs, it may occur in atypical locations including the spleen, kidney, pancreas, and gallbladder.

PGBHC is a very rare phenomenon, gallbladder cysts are often secondary to liver cysts [4]. The incidence of hydatid cyst disease has decreased for many decades. It differs depending on several factors including the presence of nomadic or semi-nomadic cattle-raising areas, economic and hygiene conditions.

In our case, we present the first case of primary gallbladder hydatid cyst with symptomatic daughter cysts in the CBD in Syria. This article aims to raise awareness about a rare presentation of a common disease as there is only a single similar case that presented with jaundice. This case is the second ever case to be treated via laparoscopic surgery, therefore, we believe that our success in treatment could be of a great help for surgeons facing similar cases.

This case report has been reported in line with SCARE 2020 criteria [5].

## 2 Case presentation

A 75-year-old male was admitted to the outpatient clinic complaining of frequent colic pain which started one month ago. other symptoms were jaundice‚ itching, vomiting, nausea, anorexia, and Insomnia.

There was no fever. urinary and bowel habits were normal.

The patient has diabetes mellitus type II treated with hypoglycemic agents and a smoker with an average of 20 cigarettes/day for 15 years.

Physical examination showed slight paleness of the skin. Neurological and cardiovascular examinations were normal.

Abdominal examination showed a positive Murphy's sign. There were no hepatomegaly or splenomegaly.

Laboratory examinations revealed an elevation in hepatic enzymes. Total bilirubin was elevated (4.4 mg/dl) and direct bilirubin was 3.2 mg/dl. Amylase and lipase enzymes were also above normal levels the differential diagnosis were cholecystitis and gallbladder stone.

Abdominal ultrasonography (USG) revealed a dilated gallbladder measuring 5 × 10 cm.

The common bile duct (CBD) was enlarged as well (1.7 cm).

The obstruction was at the end of CBD. An ERCP was conducted to treat the obstruction. Three daughter cysts were extracted from the CBD.

The patient was followed up for three weeks. hepatic enzymes regressed dramatically. bilirubin decreased as well (Total 1.7mg/dl, direct 1mg/dl)

An abdominal CT was performed, revealing a dilation in CBD (1 cm). Inside the gallbladder, a 13 × 5 cm cystic mass was detected too.

A gallstone was found as well.

Those entities applied pressure on the beginning of the CBD. The potential diagnosis was a hydatid cyst of the gallbladder.

**Fig. 1:**
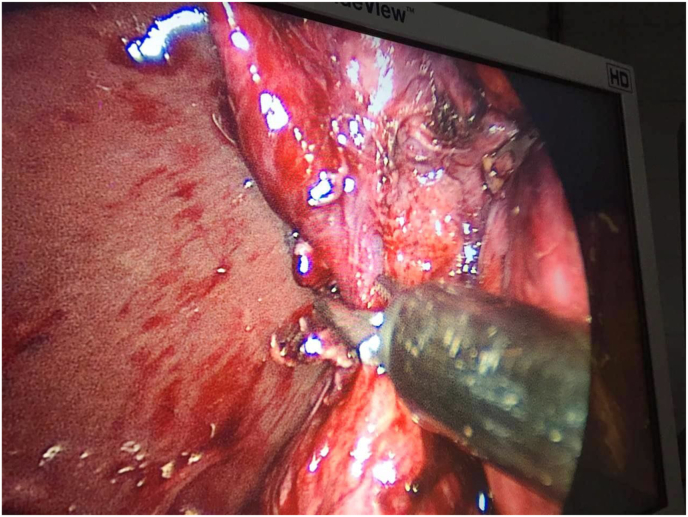
Dissection of the gallbladder by laparoscopic surgery.

**Fig. 2:**
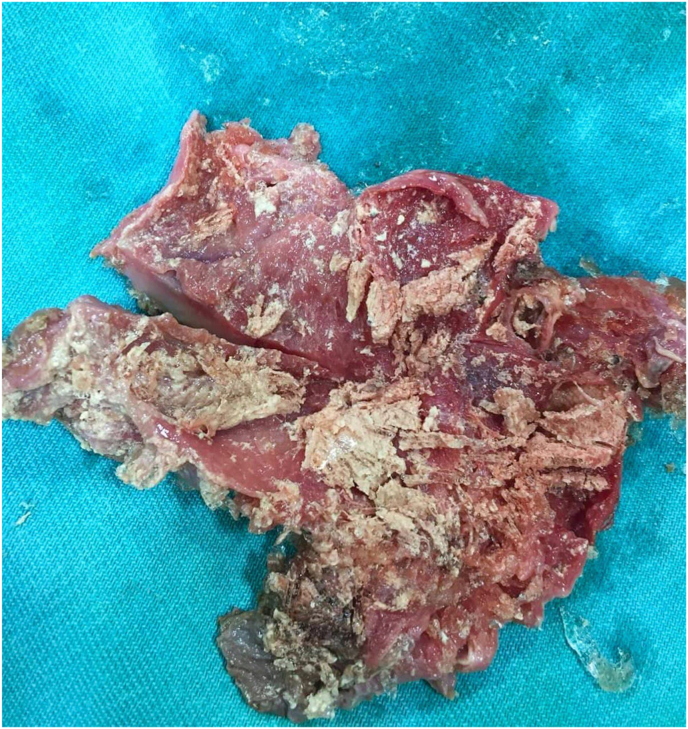
Germinal layer was recognized.

Laparoscopic surgery was performed, in which the cystic duct and artery were isolated(Fig. 1). dissection of the gallbladder revealed a ruptured hydatid cyst and the germinal layer was recognized(Fig. 2).

The patient was prescribed Albendazole after the surgery, however, it was not administered before the surgery. He was discharged on the third postoperative day.

The follow-up period was uneventful.

## 3 Discussion

Hydatid disease is a zoonotic disease described as having cysts in a certain location of the body.

This disease is caused by a parasite, a tiny worm of the genus *Echinococcus.*

Two types have been proved to have a direct causal relationship with the disease, *echinococcus multilocularis that causes alveolar echinococcosis* and echinococcus granulosis that causes *cystic echinococcosis* [6].

Dogs are the definitive host and the disease is endemic in areas of sheep and cattle raising therefore this disease is more prominent in agricultural and Mediterranean countries due to high contact with the hosts of the parasite [7,8].

Even though echinococcus granulosis is not the only type of this parasite, it is the most common type causing the disease. 95% of all *Hydatidosis* cases were due to *Echinococcus granulosis* [9].

Ingestion of this parasite's eggs causes the disease. These eggs eventually develop into six-hooklet embryos that penetrate the mucosa of the small intestine and into the portal circulation reaching the liver where filtering occurs, consequently, 70% of all cysts are found in the liver. The lung represents the second filter, with 20% of all cases [7,9]. Despite that, GBHC can be found in any part of the body including bone, cerebellum, and the thyroid, however, the incidence of extrahepatic cases is not yet known. Thus, primary hydatid cyst in the gallbladder is a very rare entity [8].

Cysts can be situated in the lumen of the gallbladder or on the external surface.

Several theories have emerged to explain the exact pathogenesis. The most well-documented theory states that the parasite reaches the gallbladder through the portal system, from the hepatic veins, and into the inferior vena cava reaching the heart. Another theory suggests the spread of the brood capsules through the biliary tract and into the cystic duct. However, this theory struggles to explain how can cysts develop on the surface of the gallbladder. Thus, another theory arose suggesting the lymphatic tract as a potential route, this theory explains both the intraluminal and the external surfaces scenarios [6,7].

**Fig. 3:**
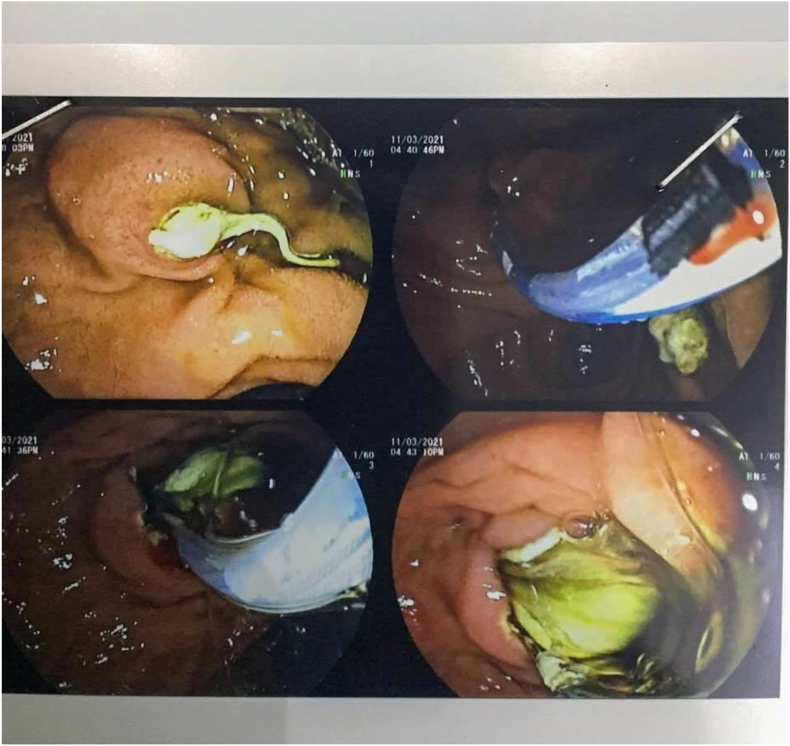
ERCP (Endoscopic retrograde cholangiopancreatography): Daughter cysts in the common bile duct.

When it comes to symptoms, it is known that cysts grow slowly over years before they cause any clinical symptoms, this is mainly true regarding relatively large organs such as the liver. However, since our case is an intraluminal cyst in the gallbladder, it takes less time compared to other locations to be clinically explicit [8]. (Fig. 3)

Abdominal pain is the main complaint present in all cases (100%), nausea and vomiting are common too (64.53%), some cases reported a positive murphy's sign (38.46%), fever is also possible (23.07%) and jaundice is the least common symptom (7.69) [4]

In our case, excluding fever, our patient presented with all symptoms mentioned in addition to anorexia and insomnia. Jaundice was caused by CBD obstruction by three daughter cysts.

Obstructive jaundice is possible in case of primary cyst rupture or migration of daughter cysts into the biliary duct.

This presentation was only reported in a single case [4]. However, in this case, jaundice and itching were one of the main presentations.

A variety of tests can be conducted to investigate *Hydatidosis*, including ultrasound, abdominal CT, and MRI. However, USG proved to be the most convenient due to its affordable nature and practicality. And in areas where the disease is endemic, it has a reasonably high diagnostic sensitivity [8]. However, other cases suggested a higher sensitivity using MRI [7].

Generally, USG is the most common (76.92%), followed by abdominal CT (46.15%), and MRI which is the least common method conducted only in (15.38%) of all cases of PGBHC [4].

**Fig. 4:**
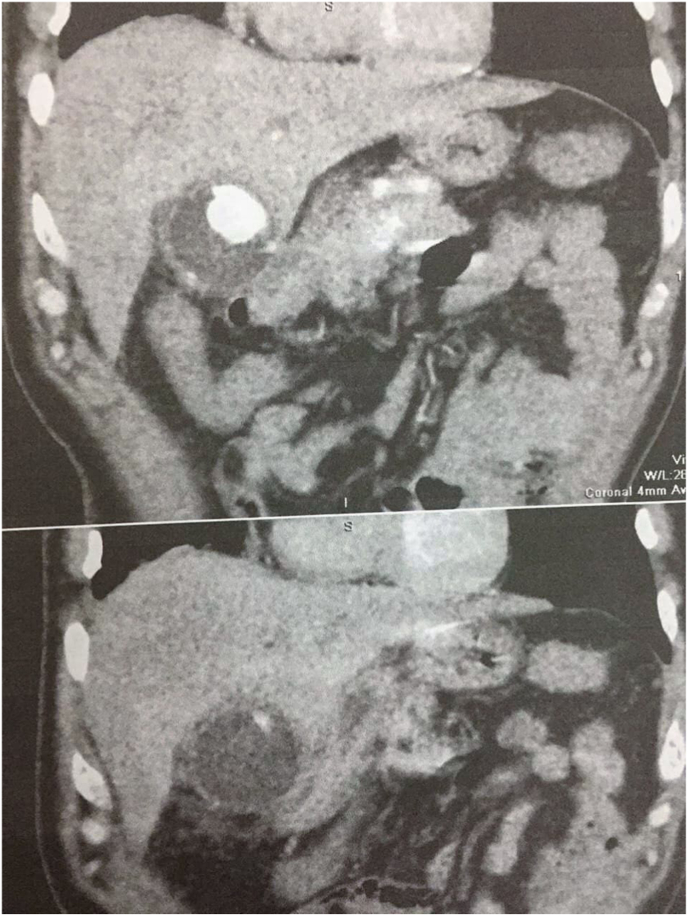
CT: Dilated gallbladder measuring 5 × 10 cm with primary hydatid cyst and calcified stone.

In our case, we conducted an USG, and following the discovery of daughter cysts, an abdominal CT was conducted, and from its findings, we were able to diagnose the disease (Fig. 4)

As per the treatment, surgery is always required [6]. open cholecystectomy is the preferred and the most common procedure. However, as surgeons are gaining more experience, laparoscopic surgery is at the present as safe as open cholecystectomy, in addition to some advantages, being less invasive for instance. There was a single case that reported laparoscopic cholecystectomy [4].

In our case, laparoscopic surgery was chosen, mainly because of the low spillage possibility and the expertise of the surgeon (Fig. 4). Overall, PGBHC is a very rare entity that is found in the agricultural areas, most likely diagnosed by USG and CT, and surgery is always the indicated upon diagnosis.

## 4 Conclusion

In conclusion, PGBHC is a rare presentation that is quite elusive to diagnose due to its non-specific symptoms and signs. However, it should be kept in mind in areas with high prevalence rates, as it could be more possible to present in these areas, even though there are only a few numbers of documented cases.

We would like also to emphasize the role of ERCP, CT, and ultrasound in establishing the diagnosis, and the role of laparoscopic surgery in treatment, as this is only the second case treated via laparoscopic approach.

## Ethical approval

An ethical approval was obtained and we can provide it upon request.

## Sources of funding

There are no funding sources.

## Author contribution

Conception and design: Ranim Kazzaz, Dana Nashed, Ghaith Izz Aldeen Sattout, Analysis and interpretation of the data: Ranim Kazzaz, Ghaith Izz Aldeen Sattout, Drafting of the article: Ranim Kazzaz, Dana Nashed, Ghaith Izz Aldeen Sattout, Nourelhuda Issa, Ahmad Aldakhil, Critical revision of the article for important intellectual content; Aghyad Kudra Danial, Ousama Bitar, All authors read and approved the final version of the manuscript.

## Declaration of competing interest

We have no Conflict of Interest.

## Registration of research studies

1. Name of the registry:
2. Unique Identifying number or registration ID:
3. Hyperlink to your specific registration (must be publicly accessible and will be checked):

## Guarantor

Aghyad Kudra Danial.

## Consent

Written informed consent was obtained from the patient for publication of this case report and accompanying images. A copy of the written consent is available for review by the Editor-In-Chief of this journal on request.

## Provenance and peer review

Not commissioned, externally peer-reviewed.

## References

[1] Ammann R.W. (1996). Parasitic diseases of the liver and intestines-Echinococcus. Gastroenterol. Clin. N. Am..

[2] Budke C.M., Deplazes P., Torgerson P.R. (2006). Global socioeconomic impact of cystic echinococcosis. Emerg. Infect. Dis..

[3] Heymann D.L. (2008).

[4] Yagnik V.D., Dawka S., Patel N. (2020). Gallbladder hydatid cyst: a review on clinical features, investigations and current management. Clin. Exp. Gastroenterol..

[5] Agha R.A., Franchi T., Sohrabi C., Mathew G., Kerwan A., Thoma A., Beamish A.J., Noureldin A., Rao A., Vasudevan B., Challacombe B. (2020). The SCARE 2020 guideline: updating consensus surgical CAse REport (SCARE) guidelines. Int. J. Surg..

[6] Krasniqi A., Limani D., Gashi-Luci L., Spahija G., Dreshaj I.A. (2010). Primary hydatid cyst of the gallbladder: a case report. J. Med. Case Rep..

[7] Noomene R., Maamer A.B., Bouhafa A., Haoues N., Oueslati A., Cherif A. (2013). Primary hydatid cyst of the gallbladder: an unusual localization diagnosed by magnetic resonance imaging (MRI). Pan Africa Med. J..

[8] Uzunoglu M.Y., Altintoprak F., Dikicier E., Zengin I., Celik A. (2016 Nov 29). Hydatid disease in the gallbladder: a rare location. J. Med. Cases.

[9] Safioleas M., Stamoulis I., Theocharis S., Moulakakis K., Makris S., Kostakis A. (2004 Oct). Primary hydatid disease of the gallbladder: a rare clinical entity. J. Hepato-Biliary-Pancreatic Surg..

