# Supplementary material for: Laparoscopic management of a primary gallbladder hydatid cyst with daughter cysts in the common bile duct: Case report
Source: Ann Med Surg (Lond). 2022 Jul 12;80:104165. doi: 10.1016/j.amsu.2022.104165 (PMC9283885; doi:10.1016/j.amsu.2022.104165)
Supplement: Multimedia component 1 [file mmc1.docx]

| **SCARE 2020 Checklist** | | | |
| --- | --- | --- | --- |
| **Topic** | **Item** | **Checklist Item Description** | **Page Number** |
| **Title** | **1** | **Laparoscopic management of a primary gallbladder hydatid cyst with daughter cysts in the common bile duct: Case Report** | 1 |
| **Key Words** | **2** | Gallbladder, Primary hydatid cyst, laparoscopic management, Common bile duct, Daughter cyst, Case report.  . | 1 |
| **Abstract** | **3a** | Introduction and Importance   - One of the very rare presentation of hydatid cysts, that is treated with laparoscopic surgery | 1 |
|  | **3b** | Case Presentation   - colic pain, jaundice, itching, vomiting, nausea, insomnia. | 1 |
|  | **3c** | Clinical Findings and Investigations   - Positive murphy’s sign, and elevated AST and ALT. Total bilirubin was also elevated, USG, CT, ERCP. The overall diagnosis was PGBHC | 1 |
|  | **3d** | Interventions and Outcome   - Laparoscopic was selected because of our patient’s age and because the cyst was already ruptured. - Total recovery after 1 month follow-up. | 1 |
|  | **3e** | Relevance and Impact   - a rare presentation - ERCP, CT, and ultrasound in establishing the diagnosis - the role of laparoscopic surgery in treatment | 1 |
| **Introduction** | **4** | Background: a brief definition of Hydatid cysts disease and the related biostatistics were mentioned  Rationale  We would like to emphasize of the role of ERCP, CT and ultrasound in establishing diagnosis, and the role of laparoscopic surgery in treatment, as this is only the second case treated via laparoscopic approach.  primary hydatid cyst in the gallbladder is a very rare entity and its incidence is not yet known  This disease is endemic in Mediterranean countries due to the contact with the host of the parasite.  Guidelines and Literature   - [6] [7] - Were mentioned in the main document | 1  1  2 |
| **Patient Information** | **5a** | Demographic Details   - Male 75 years old farmer | 2 |
|  | **5b** | Presentation  Frequent colic pain  Self-presentation to the outpatient clinic | 2 |
|  | **5c** | Past Medical and Surgical History  Medical records of the patient included a diagnosis of diabetes mellitus II. | 2 |
|  | **5d** | Drug History and Allergies  The patient is a type II diabetic treated with diabetic treated with hypoglycaemic.  The patient had no familial, genetic or psychological history | 2 |
|  | **5e** | Family history:  Non significant.  The patient is a smoker with an average of 20 cigarettes/day. | 2 |
| **Clinical Findings** | **6** | The abdominal examination showed positive Murphy's sign | 2 |
| **Timeline** | **7** | frequent colic pain which started one month ago, other symptoms were jaundice‚ itching, vomiting, nausea, and anorexia. Insomnia | 2 |
| **Diagnostic Assessment and Interpretation** | **8a** | Diagnostic Assessment   - Abdominal ultrasonography - Laboratory AST and ALT). total bilirubin . Amylase and lipase - Imaging (abdominal ultrasound, CT). - Invasive (ERCP). | 2 |
|  | **8b** | Diagnostic Challenges   - MRI imaging was not accessible in the hospital due to financial issues. | 2 |
|  | **8c** | Diagnostic Reasoning   - Cholecystitis, gall bladder stone | 2 |
|  | **8d** | Prognostic Characteristics  Not applicable | - |
| **Intervention** | **9a** | Pre-Operative Patient Optimisation | - |
|  | **9b** | Surgical Interventions   - laparoscopic surgery . - The patient received medical treatment with Albendazole after the surgery | 2 |
|  | **9c** | Specific Details regarding Interventions  In our case the surgery of choice was laparoscopic, mainly because of the low spillage possibility due to the previous cyst rupture and the laparoscopic expertise of the surgeon  Describe the rationale behind the treatment offered, how it was performed and time to intervention.  this is only the second case treated via laparoscopic approach. | 4  3 |
|  | **9d** | Operator Details and Setting of Intervention  Dr. Aghyad Kudra Danial Attending general surgeon  Aleppo university hospital | - |
|  | **9e** | Deviation from Initial Management Plan   - There were no changes in the planned intervention. | - |
|  | **9f** | Post-intervention considerations e.g. post-operative instructions and place of care:  There were none |  |
| **Follow-Up and**  **Outcomes** | **10a** | The patient was followed up after the operation for several months and no complications were reported.   - Secondary care - clinical examination | 1  1 |
|  | **10b** | Intervention Adherence and Compliance   - none | - |
|  | **10c** | Outcomes   - We had full recovery with no complications. | 1 |
|  | **10d** | Complications and Adverse Events   - There were no adverse events | - |
| **Discussion** | **11a** | Strengths   - A very rare presentation with a with a rare intervention - A gastroenterologist preformed the ERCP | 1  2 |
|  | **11b** | Weaknesses and Limitations   - The difficulty of obtaining an MRI image | 2 |
|  | **11c** | Relevant Literature   - Similar cases were discussed and the approaches were compared | 3 |
|  | **11d** | - Our conclusion comes from the advantage of the low invasive nature of the laparoscopic technique and the favourable outcomes during remission | 4 |
|  | **11e** | Take-Away Lessons  it should be kept in mind in areas with high prevalence rates, as it could be more possible to be present in these areas, even though there are only a few numbers of cases documented. | 4 |
| **Patient Perspective** | **12** | - The patient reported a good relief of symptoms, with no surgery related complaints. | - |
| **Informed Consent** | **13** | - Written informed consent was obtained from the patient for publication of this case report and accompanying images. A copy of the written consent is available for review by the Editor-In-Chief of this journal on request | 4 |
| **Additional Information** | **14** | - There was no conflict of interest - Contributions were mentioned | 4 |
| **Clinical Images and Videos** | **15** | - Where relevant and available, include clinical images to help demonstrate the case pre-, peri-, and post-intervention (e.g. radiological, histopathological, patient photographs, intraoperative images). - Where relevant and available, include a link (e.g. Google Drive, YouTube) to the narrated operative video can be included to highlight specific techniques or operative findings. - Ensure all media files are appropriately captioned and indicate points of interest to allow for easy interpretation. | 2  3  4 |
| **Referencing the Checklist** | **16** | - Include reference to the SCARE 2020 publication by stating: ‘This case report has been reported in line with the SCARE Criteria. | 1 |
